# Beneficial effects of low dose radiation in response to the oncogenic KRAS induced cellular transformation

**DOI:** 10.1038/srep15809

**Published:** 2015-10-30

**Authors:** Rae-Kwon Kim, Min-Jung Kim, Ki Moon Seong, Neha Kaushik, Yongjoon Suh, Ki-Chun Yoo, Yan-Hong Cui, Young Woo Jin, Seon Young Nam, Su-Jae Lee

**Affiliations:** 1 Department of Life Science, Research Institute for Natural Sciences, Hanyang University, Seoul, Korea; 2 Laboratory of Radiation Exposure and Therapeutics, National Radiation Emergency Medical Center, Korea Institute of Radiological and Medical Sciences, Seoul, Korea; 3 Radiation Health Institute, Korea Hydro and Nuclear Power Co. Ltd, Seoul, Korea

## Abstract

Recently low dose irradiation has gained attention in the field of radiotherapy. For lack of understanding of the molecular consequences of low dose irradiation, there is much doubt concerning its risks on human beings. In this article, we report that low dose irradiation is capable of blocking the oncogenic *KRAS*-induced malignant transformation. To address this hypothesis, we showed that low dose irradiation, at doses of 0.1 Gray (Gy); predominantly provide defensive response against oncogenic *KRAS* -induced malignant transformation in human cells through the induction of antioxidants without causing cell death and acts as a critical regulator for the attenuation of reactive oxygen species (ROS). Importantly, we elucidated that knockdown of antioxidants significantly enhanced ROS generation, invasive and migratory properties and abnormal acini formation in *KRAS* transformed normal as well as cancer cells. Taken together, this study demonstrates that low dose irradiation reduces the *KRAS* induced malignant cellular transformation through diminution of ROS. This interesting phenomenon illuminates the beneficial effects of low dose irradiation, suggesting one of contributory mechanisms for reducing the oncogene induced carcinogenesis that intensify the potential use of low dose irradiation as a standard regimen.

In recent years, even though high ionizing radiations (IR) effect on cancer cells such as direct chromosomal DNA damage (double-stranded breaks), chromosomal aberrations or genetic mutations for alteration of malignant phenotype or indirect DNA damage caused by the free radicals formed due to ionization resulting have been broadly explored. However, the biological aspects of low dose irradiation remained controversial. Once the cancer is diagnosed at a progressive stage, radiation is one of the standard treatments for the disease. Recently, radiotherapy has attracted the growing interest in low dose irradiation whether cancer risk ensues after exposure to low dose irradiation in the dose range under certain threshold level. In contrast to our knowledge, as harm to normal tissues around a tumor is unavoidable in radiotherapy, a high dose of IR is often fractionated to reduce the harmful effects of radiotherapy. A delicate balance of highest dose application to the tumor and minimal dose exposure to healthy tissue has always to be taken into consideration when applying radiation techniques and dose fractionating systems^1^. In the earth’s atmosphere, many nuclear industry workers are exposed to low or fractionated dose of radiation. Also epidemiological statistics have shown that residents of high-background radiation in India, China, USA, Japan and many other parts of the world have lower cancer death than those living in areas with a normal background radiation environment^2^,^3^. These outcomes could be expected mainly due to the reason because low dose irradiation correlates with immune enhancement^4^. Low dose irradiation activated immune response ensues through the stimulation of both antigen-presenting cells and T lymphocytes, assisting intercellular reactions within the immunological synapse^5^. Still, the particular health risks from exposure to low dose irradiation persist unclear. These days, LNT (linear no-threshold) dose model is widely accepted to predict the effects of low dose irradiation by linear extrapolation from those detected at maximum doses^6^. Likewise, there seems to be no safe radiation dose, as even very low doses of radiation can show some biological effects^7^. Several studies contradict the validity of this concept by exhibiting risk diminution, i.e., radiation hormesis, of cancer death in populations exposed to low-dose radiation^8^. However, underlying molecular mechanisms regarding the biological effect of low dose irradiation in human welfare remained largely obscure. Ensuing research on this topic became a hot issue.

In our earlier work, we showed that oncogene *KRAS* (Kirsten rat sarcoma viral oncogene homolog) induces ROS generation through activation of NADPH oxidase, which is a severe regulator for the *KRAS* induced cellular transformation^9^,^10^. *KRAS* are small GTPase binding proteins that acts as a plasma membrane localized molecular switch and regulates many signaling pathways linked with cell proliferation and survival^11^,^12^. Aberrant activation in the *KRAS* gene has significant effects in tumor progression, which rely on the cells and tissues intricate^13^. It is noteworthy that as a well-known oncogene, *KRAS* is mostly activated and overexpressed in more than 35–40% malignancies, including breast cancer. Moreover, we also reported that *KRAS* promoted mesenchymal features in basal type breast cancer cells^14^. Therefore, we selected the breast cancer cell line to investigate the effect of low dose irradiation on malignant phenotype. Current therapies treating this cancer progression limit cancer development but still sustain the most common reason of cancer-related death among women worldwide^15^. This cancer therapy usually involves surgery, chemotherapy or radiation therapy. In context to radiology, high dose radiation is known to suppress the immune system for cancer destruction^16^,^17^. Conversely, many lines of evidences from the last decade have exposed that low dose irradiation remarkably play critical role in cancer therapy whereas molecular mechanisms that initiate this phenomenon have not been identified so far^18^. Experimental studies have proven that phenomena such as adaptive response and cell–cell communication also responsible for improved therapeutic gain using low dose irradiation^19^,^20^,^21^. Variations in innate immune response and suppression of metastasis subsequent have been stated with the low dose irradiation also^16^,^22^. Furthermore, low dose irradiation has been revealed to enhance the efficacy of chemotherapeutic drugs^23^.

Therefore we sought to develop a means by which efficiently *KRAS* induced malignant transformation could be suppressed using low dose irradiation so that simultaneously dosing with anticancer drug might provide the safe therapeutic effect that has so far remained obscure. The data presented in this paper indicate that low dose irradiation promotes a reduction of malignant phenotype in oncogenic transformed cells. Moreover, down-regulation of antioxidants confirms that these are key factor involved in low dose irradiation reduced malignant phenotype in transformed cells.

## Results

### Low-dose irradiation provides defensive response against malignant phenotype induced by KRAS

**Figure 1:**
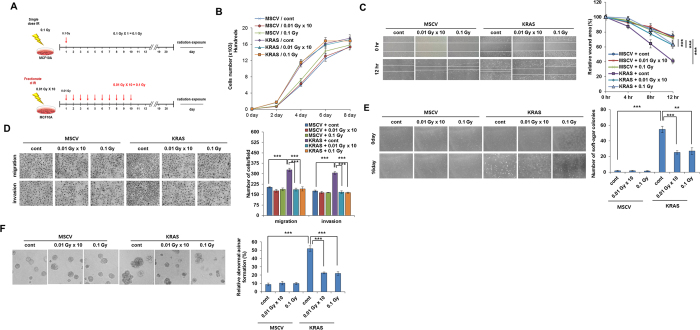
Low-dose irradiation provides defensive response against malignant phenotype induced by KRAS (**A**) Experimental scheme of low-dose irradiation exposure to cells. Cells were either treated with 0.1 Gy at once or exposed with 0.01 Gy for 10 times (accumulative dose of 0.1 Gy). (**B**) Growth curve of low-dose irradiation treated MCF10A cells upto 8 days which were transfected with MSCV- *KRAS* and control empty MSCV vector. (**C**) Images and graphical representation of wound healing assay of MSCV- *KRAS* or control empty MSCV vector transfected low-dose irradiated-MCF10A cells. (**D**) Images and graphical representation of migration and invasion assay of MSCV-*KRAS* and control empty MSCV vector transfected low-dose irradiated-MCF10A cells. (**E**) Images and graphical representation of soft agar assay of MSCV-*KRAS* and control empty MSCV vector transfected low-dose irradiated-MCF10A cells. (**F**) Images and graphical representation of acini formation assay of MSCV-*KRAS* and control empty MSCV vector transfected low-dose irradiated-MCF10A cells. Error bars represent the ± SD (n = 3). Student’s *t*-test was performed, and the significance is designated as *p < 0.05, **p < 0.01, and ***p < 0.001.

To explore a potential role for low dose irradiation as a breast cancer suppressor, we conducted this study with the normal breast epithelial MCF10A cell line. To examine the fractionated or single low dose irradiation effect on transformed breast epithelial cells (Fig. 1A); we transduced the breast normal cells with overexpression of *KRAS* gene. When cell growth was investigated, we found that treatment of low dose irradiation did not block the cell proliferation in KRAS transformed MCF10A cells (Fig. 1B). We have performed Annexin-V/PI staining on low dose irradiated MCF10A cells. But there was no significant cell death caused by either *KRAS* or low dose irradiation (supplementary figure S1). On the basis of this data, we concluded that ectopic expression of KRAS does not cause significant cell death and LDR does not enhance or reduce cell death in the normal cells (Fig. S1). Because transformed cells have invasive and migratory properties, we next examined whether low dose irradiation contributes to defend against these malignant features in transformed MCF10A cells. Here, we found that low dose irradiation inhibited the migratory and invasive properties in *KRAS* transformed MCF10A cells (Fig. 1C,D). Another feature of the transformed or malignant phenotype is the competence for anchorage-independent proliferation^24^. Similarly, treatment with low dose irradiation also blocked *KRAS* enhanced colonization capacity in *KRAS* transformed MCF10A cells when compared with un-irradiated cells in soft agar (Fig. 1E). As it is already known that ontogenetically transformed mammary epithelial cells form large, non-polarized, undifferentiated colonies (acinar) without lumina when grown in matrigel^25^. Next, to confirm more the efficacy of low dose irradiation, we further tested this *ex-vivo* model on the morphology of structures formed by *KRAS* transformed MCF10A cells^26^. We formed the three-dimensional (3D) acinar structures by plating MCF10A cells as single cells in collagen mixed matrigel. Microscopic analyses of acini formation revealed that low dose irradiation blocked the *KRAS* induced abnormal acini formation in transformed MCF10A cells (Fig. 1F). Interestingly, we also observed the similar effects of low dose irradiation in *KRAS* transformed normal embryonic lung fibroblast WI38 cells also (Fig. S2). Low dose irradiation was also shown to be effective at very low or intermediate doses too; however it was not much significant (Fig. S3). Taken together these results indicated that fractionated or single low dose irradiation dose can reduce the malignant phenotype in ontogenetically transformed cells.

### Low-dose irradiation counteracts KRAS induced ROS levels

**Figure 2:**
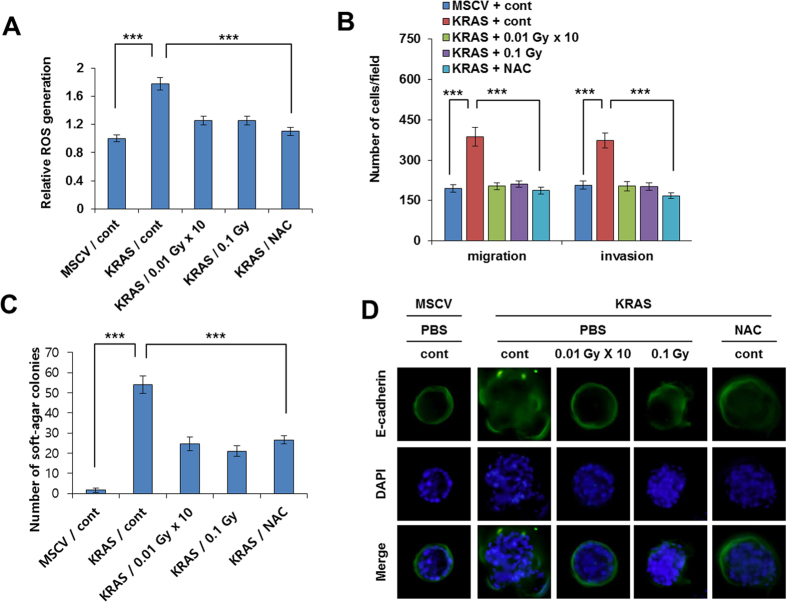
Low-dose irradiation decreases KRAS-induced ROS production, thereby inhibiting KRAS-induced transformation. (**A**) Relative ROS generation of low-dose irradiated-MCF10A cells which were transfected with MSCV-*KRAS* and control empty MSCV vector, as measured by FACS. (**B**) Migration and invasion assay of MSCV-*KRAS* and control empty MSCV vector transfected low-dose irradiated-MCF10A cells. (**C**) Soft agar assay of low-dose irradiated-MCF10A cells which were transfected with MSCV-*KRAS* and control empty MSCV vector. (**D**) Cellular localization of E-cadherin (green) in low dose irradiated-MCF10A cells which were transfected with MSCV-*KRAS* or control empty MSCV vector. NAC (N-acetyl L-cysteine) was used as a ROS inhibitor to compare low dose irradiation effect as positive control. Error bars represent the ± SD (n = 3). Student’s *t*-test was performed, and the significance is designated as *p < 0.05, **p < 0.01, and ***p < 0.001.

Since we hypothesized that reactive oxygen species (ROS) are involved in *KRAS* induced cellular oncogenic transformation [10], we next sought to determine the ROS levels in the low dose irradiated *KRAS* overexpressed MCF10A cells. Intriguingly, we observed low dose irradiation decreased the *KRAS* induced ROS levels in the transformed MCF10A cells (Fig. 2A and Fig. S4). N-acetyl L-cysteine (ROS inhibitor) is used here as a ROS inhibitor (positive control). Given the extensive impact of epithelial–mesenchymal transition (EMT) in cancer progression^27^, we also checked migration and invasion properties of *KRAS* overexpressed MCF10A cells. Our data revealed that low dose irradiation treatment block the *KRAS* induced migration and invasion in transformed MCF10A cells similar to NAC (Fig. 2B). Also low dose irradiation reduced the *KRAS* induced colony forming ability of MCF10A cells in soft agar similar to NAC (Fig. 2C). As loss of E-cadherin expression is the first step towards the initiation of EMT process; we next examined the expression of E-cadherin after low dose irradiation exposure. Notably, low dose irradiation also recovered the E-cadherin at cell surface where it is localized, in *KRAS* transformed MCF10A cells similar to NAC (Fig. 2D). These finding suggests that low dose irradiation play a critical role in failure of ROS generation and EMT program induced by *KRAS*.

### Function of glutathione peroxidase contributes the low dose irradiation induced ROS destruction for EMT failure in transformed normal cells

**Figure 3:**
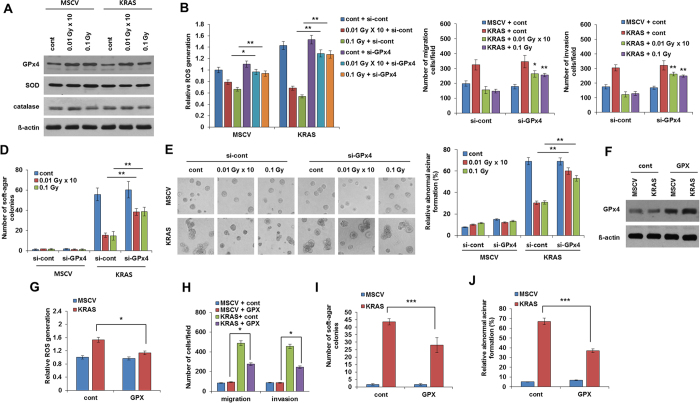
Low-dose irradiation decreases KRAS-induced ROS production through induction of antioxidant, GPx4 (**A**) Western blot analysis of antioxidants using cell lysate from low dose irradiated-MCF10A cells which were transfected with MSCV-*KRAS* and control empty MSCV vector. (**B**) Relative ROS generation of low dose irradiated-MCF10A cells which were transfected with control siRNA and siRNA against GPx4. (**C**) Migration and invasion assay of MSCV control and MSCV *KRAS* transfected low dose irradiated-MCF10A cells after blocking GPx4 expression using siRNA targeting. (**D**) Graphical representation of number of soft agar colonies of MSCV control and MSCV *KRAS* transfected low dose irradiated-MCF10A cells after targeting of GPx4 expression using siRNA. (**E**) Images and graphical representation of acini formation assay of MSCV control or MSCV-*KRAS* transfected low dose irradiated-MCF10A cells after targeting of GPx4 expression using siRNA. (**F**) Western blot of GPx4 from lysates of MCF10A cells which were transformed with MSCV control or MSCV-*KRAS* and further overexpressed by GPX. (**G**) Relative ROS generation in *KRAS* transformed MCF10A cells after overexpressed by GPX. (**H**) Migration and invasion assay in *KRAS* transformed MCF10A cells after overexpressed by GPX. (**I**) Number of soft agar colonies in *KRAS* transformed MCF10A cells after overexpressed by GPX. (**J**) Acini formation in *KRAS* transformed MCF10A cells after overexpressed by GPX. Error bars represent the ± SD (n = 3). Student’s *t*-test was performed, and the significance is designated as *p < 0.05, **p < 0.01, and ***p < 0.001.

Antioxidant plays a dynamic role in neutralization of excess ROS. In agreement with above results that low dose irradiation abolishes *KRAS* induced ROS levels; we proceeded to investigate the effect of low dose irradiation on antioxidants levels in *KRAS* overexpressed MCF10A cells. To this end, we explored the enzymatic antioxidants such as superoxide dismutase (SOD), catalase (CAT) and glutathione peroxidase (GPx4). Here, we found that GPx4 was enhanced after low dose irradiation treatment in *KRAS* transformed MCF10A cells when compared to control while SOD and CAT both remain unchanged (Fig. 3A). To further confirm the potential involvement of GPx4 in low dose irradiation for ROS counteraction, we next investigated whether GPx4 effect ROS generation in transformed MCF10A cells through low dose irradiation. Notably, siRNA-mediated down-regulation of GPx4 recovered the ROS generation in *KRAS* transformed MCF10A cells (Fig. 3B). Moreover, down-regulation of GPx4 also increased the *KRAS* induced invasion and migration in transformed cells (Fig. 3C and Fig. S5). Similarly, when soft agar colony formation and acini formation was analyzed using si-GPx4, we observed that down-regulation of GPx4 enhanced the *KRAS* induced colony and acini formation in transformed MCF10A cells, which is a characteristic feature of malignant breast phenotype (Fig. 3D,E and Fig. S6). Strikingly, overexpression of GPx4 also seems to be affecting the ROS induction level, migration and invasion, colony and acini formation in *KRAS* transformed cells (Fig. 3F–J). Collectively these results indicate that GPx4 is the main factor in regulating low dose irradiation decreased malignant phenotype in transformed MCF10 A cells.

### Low dose irradiation induced catalase promotes the malignant phenotype attenuation in cancer cells

**Figure 4:**
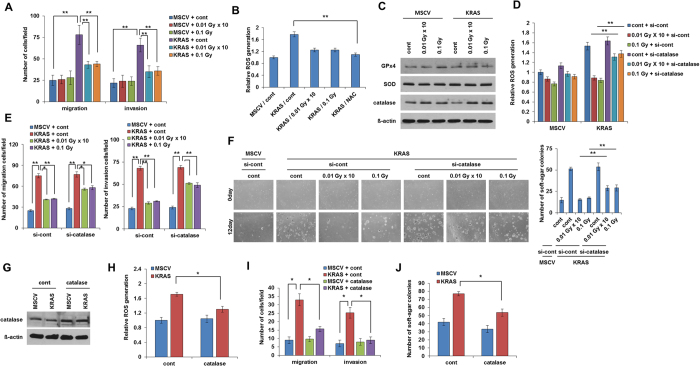
Low dose irradiation-induced catalase attenuates KRAS-induced migration and invasion in breast cancer cells. (**A**) Migration and invasion assay of low dose irradiation exposed MCF7 cells which were transformed with MSCV-*KRAS* or control empty MSCV vector. (**B**) Relative ROS generation in low dose irradiated-MCF7 cells which were transformed with MSCV-*KRAS* or control empty MSCV vector. NAC was used here as a ROS inhibitor cells as positive control. (**C**) Western blot analysis of antioxidants using cell lysate from low dose irradiated-MCF7 cells transfected with control empty MSCV vector or MSCV-*KRAS*. (**D**) Relative ROS generation in MCF7 cells transformed with control MSCV vector or MSCV-*KRAS* and was transfected with control siRNA or siRNA against catalase. (**E**) Migration and invasion assay of low dose irradiated-MCF7 cells transformed with control MSCV vector or MSCV- *KRAS* which were transfected with control siRNA or siRNA against catalase. (**F**) Images and graphical representation of low dose irradiated-MCF7 cells transformed with control MSCV vector or MSCV-KRAS which were transfected with control siRNA or siRNA against catalase. (**G**) Western blot of catalase from lysates of MCF7 cells which were overexpressed by catalase. (**H**) Relative ROS generation in MCF7 cells which were transfected with MSCV control or MSCV*-KRAS* and overexpressed by catalase. (**I**) Migration and invasion of MCF7 cells which were transfected with MSCV control vector or MSCV-*KRAS* and further overexpressed by catalase. (**J**) Number of soft agar colonies in MCF7 cells which were transfected with MSCV control vector or MSCV-*KRAS* and further overexpressed by catalase. Error bars represent the ± SD (n = 3). Student’s *t*-test was performed, and the significance is designated as *p < 0.05, **p < 0.01, and ***p < 0.001.

To strong our observations more in detail, we also performed our study in *KRAS* overexpressed malignant MCF7 cells. In line with above data, treatment of low dose irradiation also attenuated the migration and invasion of MCF7 cells (Fig. 4A). Of note, ROS level was decreased by the low dose irradiation in *KRAS* overexpressed MCF7 cells (Fig. 4B). Western blot analysis confirms that only catalase was increased through low dose irradiation in *KRAS* overexpressed MCF7 cells while GPx4 and SOD remain unaffected (Fig. 4C). As expected, knockdown of CAT significantly recovered the ROS generation (Fig. 4D) and enhanced migration and invasion, colony formation in low dose irradiation treated *KRAS* overexpressed breast cancer cells (Fig. 4E,F). Furthermore, overexpression of CAT leads to the attenuation of ROS, migration and invasion, colony formation in overexpressed MCF7 breast cancer cells (Fig. 4G–J). Taken together, these results indicate that low dose irradiation induced attenuation of ROS is critical phenomenon for the suppression of malignant phenotype in oncogenic transformed cells.

## Discussion

Recently radiation is extensively used for cancer therapy but high dose of radiation can also have harmful effects on normal counterparts, by this means causing unwanted many harmful effects. On the other hand, low dose irradiation can be largely beneficial to living organisms through immune response enrichment and efficacy of chemotherapeutic agents^17^,^28^, but almost no information is available the possible contribution of low dose irradiation in enhancing radiation-induced malignancy reduction and its consequential mechanism. It has been reported that a small fraction of IR (<0.4 Gy) delivered before larger fraction (>0.4 Gy) resulting to insignificant increase in cancer cell killing *in vitro*^29^. Conversely, some evidence shows that three small fractions (0.4 Gy each day) failed to improve the result in glioblastoma xenografts *in vivo* systems^30^. Many factors may influence that low dose irradiation exposure has been also associated to an increased cancer risk^31^. On the other hand, low dose irradiation has been known to regulate wide variety of cellular processes such as aging, survival^32^,^33^ and involved in apoptosis of many types of cancer cells as well as proliferation^34^. Interestingly, repair of double stranded breaks, cell cycle checkpoint controls and mitotic cell death were also observed after low dose irradiation. Moreover, *in vitro* experiments have proven that low dose irradiation below a dose rate of 20 mGy does not induce DSBs nor phosphorylation of p53 gene in normal human fibroblasts^35^,^36^. However, the LNT model has been accepted for estimating the cancer risk from ionizing radiation, even the epidemiological study could not disclose the certain consequences of the radiation induced effects. To find out about these uncertainties, molecular mechanisms of radiation-induced carcinogenesis should be investigated. In the context of inflammation, high dose radiation can have unwanted adverse effect on normal cells during cancer therapy. Whereas, recently Hong *et al.* reported that LDR successfully inhibited the inflammation by blockage of catenin signaling pathway in chondrocytes without causing any side effects at doses below than 1 cGy^37^.

**Figure 5:**
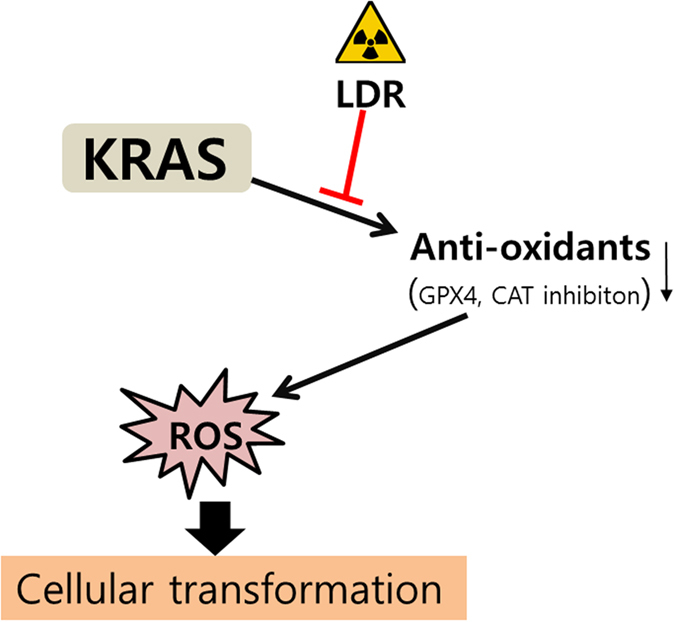
Proposed mechanism for the effect of low dose radiation (LDR) on KRAS induced cellular transformation.

Generally human cancers (carcinomas), including breast cancer, are initiated by the malignant transformation of epithelial cells^38^. Acini formation usually reiterate in three dimensional cultures during morphogenesis in non-transformed mammary epithelial cell lines for such as MCF10A^39^. This feature characterizes a significant tool to illustrate the biological events of oncogenes. Many reports suggested that several oncogenes can have diverse disturbing effects on acini morphogenesis and induce perturbations in the cell colonies, which are detected by pathologists in neoplastic lesions^38^. However, these findings were restricted to cells with some specific oncogene expression. Based on this knowledge, in this study we demonstrate that low dose irradiation represents an efficient interference to block the recruitment of large, non-polarized, undifferentiated colonies i.e. acini formation from epithelial cells as well as anchorage-independent growth in transformed mammary cells. Ionizing irradiation elicited the reduction of *KRAS* induced malignant phenotype by expressing antioxidants. GPx4 and CAT activity by low dose irradiation were exclusively responsible for the subsequent malignancy reduction. The critical role of these antioxidants was further emphasized by our finding that knockdown of GPx4 and CAT also blocked malignant characteristics such as migration and invasion. Thus, local low dose irradiation may also represent a promising adjuvant strategy for radiation based cancer therapy. In previous studies, we have shown that ROS is essential for *KRAS* induced efficient cellular transformation^10^. Many reports has been suggested that low dose irradiation involves in the different biological activities like DNA damage repair, immune response, apoptosis and antioxidant activity *in vitro* as well as *in vivo*. In agreement with these studies, we applied low dose irradiation in *KRAS* transformed cells and found that antioxidants induction is crucial in low dose irradiation induced reduction in malignant phenotype. Interestingly, we noted that overexpression of antioxidants decreased the ROS generation, migration and invasion in *KRAS* transformed cells. These findings suggest that ROS destruction was critical for suppression of malignant phenotype using low dose irradiation (Fig. 5).

Collectively, our results represent the first time evidence that very low dose (0.1 Gy) of ionizing radiation reduces the *KRAS* induced malignant cellular transformation in mammary cells following the up-regulation of antioxidants levels (GPx4 and CAT) and decreases the ROS generation. There was no specific cell death was observed in normal mammary cells by fractionated and single dose of low dose irradiation even through in the presence of *KRAS* oncogene. In all tested conditions, both fractionated as well as single dose of low dose irradiation was capable in malignant phenotype reduction. Since radiotherapy differs considerably among persons on the basis of amount of dose given and cell types, development of radiation dose that are effective at low doses will help in the individual’s treatment strategy using low dose irradiation.

## Materials and Methods

### Chemical antibodies

Polyclonal antibody to E-cadherin was obtained from BD Transduction Laboratory (Seoul, Korea). Polyclonal antibodies to GPx4 and Catalase were purchased from Abcam (Cambridge, UK). Polyclonal antibody to SOD was obtained from Enzo Life Sciences (New York, USA). 4, 6-Diamidino-2-phenylindole (DAPI), and monoclonal antibodies to β-actin were obtained from Sigma. Anti-mouse Alexa Fluor 488 and anti-rabbit Alexa Fluor 488 were purchased from Invitrogen.

### Cell Culture and transfection

Normal human breast epithelial cell line MCF10A and breast cancer cell line MCF-7 were established from the American Type Culture Collection (Manassas, VA). Cells were cultured in a humidified 5% CO_2_ atmosphere at 37 °C. The MCF10A was maintained in DMEM/F-12 medium supplemented with 5% heat-inactivated horse serum (Invitrogen), 10 μg/ml insulin, 20 ng/ml EGF, 0.1 μg/ml cholera toxin, 0.5 μg/ml hydrocortisone, penicillin (100 units/ml), and streptomycin (100 μg/ml). MCF7 cells were grown in minimum Eagle’s medium supplemented with 10% fetal bovine serum, penicillin (100 units/ml), and streptomycin (100 g/ml). siRNA duplexes were introduced into cells using Lipofectamine-2000 reagent (Gibco Invitrogen Corp) according to the procedure recommended by the manufacturer. Cells were harvested after 48 h for subsequent experiments. All siRNA were purchased from Genolution Co. Ltd. (Korea, Seoul).

### Irradiation

Cells were plated in 100 mm dish and irradiated at room temperature with a 137Cs laboratory γ-irradiator (LDI-KCCH 137, Seoul, Korea) at a dose rate of 0.1 Gy/min for the time required to apply a prescribed dose. For fractionated radiation of 0.1 Gy, cells were irradiated on five consecutive days in a week during two weeks to establish 0.01 Gy × 10 fractionated radiation cells. The single dose radiation was applied at the same time as the last dose of fractionated radiation.

### Transduction

Oncogenic *KRAS* (G13D) was cloned into retroviral vector MSCV. For retrovirus production, H29D cell line was cultured in DMEM (Invitrogen) supplemented with 10% fetal bovine serum, 2 mmol/liter GlutaMAX (Invitrogen), 50 units/ml penicillin/streptomycin, 1 μg/ml tetracyclin, 2 μg/ml puromycin, and 0.6 mg/ml G418 sulfate (Calbiochem) and transfected with MSCV or MSCV-*KRAS* using the Lipofectamine 2000 reagent (Invitrogen). After 48 h of the subsequent transfection, viral supernatant was harvested and passed through a 0.45-μm filter, and the viral supernatant was frozen at −80 °C. This supernatant was used for infection to the mammary cells after adding 4 μg/ml Polybrene (Sigma).

### Quantification of cell death

Cell death was measured by FACS analysis using propidium iodide (1:1000) staining. Cells were harvested by trypsinization, washed in PBS, and then incubated in propidium iodide (50 ng/ml) for 5 min at room temperature. Cells (10,000 per sample) were analyzed by BD FACSCalibur, using Cell Quest software.

### Invasion and migration assays

Cells (2 × 10^4^ cells/well) were suspended in 0.2 ml of growth medium for invasion and migration assays. For invasion assay, the cells were loaded in the upper well of the Transwell chamber (8 μm pore size; Corning coaster) that was pre-coated with 10 mg/ml growth factor-reduced Matrigel (BD Biosciences) with the lower well filled with 0.8 ml of growth medium. After incubation for 48 h at 37 °C, non-invaded cells on the upper surface of the filter were removed with a cotton swab, and migrated cells on the lower surface of the filter were fixed and stained with a Diff-Quick kit (Fisher-scientific) and photographed (magnification × 20). Invasiveness was determined by counting cells in five microscopic fields per well, and the extent of invasion was expressed as an average number of cells per microscopic field. Cells were imaged by phase contrast microscopy (Leica Microsystems, Bannockburn, IL). For migration assay, we used the chambers with control inserts that contained the same type of membrane but without the Matrigel coating (one chamber per well of a 24-well plate). 2 × 10^4^ cells in 0.2 ml of growth medium were added to the apical side of each insert, and 0.8 ml of growth medium was then added to the basal side of each insert. The inserts were processed as described above for the invasion assay.

We also analyzed migration by wound healing assays. To this end, cells were plated in 60 mm culture dish and grown to 80% confluence in complete medium. A ‘wound’ was made by scraping with a P200/yellow pipette tip in the middle of the cell monolayer. Floating cells were removed by washing with phosphate-buffered saline and fresh complete medium was added. Cells were incubated at 37 °C for 24 h. Cells were imaged with by phase-contrast microscopy (Leica Microsystems).

### Soft agar colony formation assay

To examine anchorage independent growth, a cell suspension (2 × 10^4^ cells) was suspended in 0.4% agar in growth medium and seeded in triplicate on 60-mm dishes pre-coated with 0.8% agar in growth medium and incubated at 37 °C with 5% CO_2_. After 12–24 days, colonies were photographed and counted in four randomly chosen fields and expressed as means of triplicates, representative of two independent experiments.

### Morphogenesis assay

To observe the abnormal acini formation, MCF10A was suspended in culture medium. Briefly, eight-well cell culture slide (SPL) were coated with 50 μl of Matrigel per well. Then, 5 × 10^3^ cells were plated per well in assay medium containing a final concentration of 2% Matrigel. Assay medium containing 2% Matrigel was replaced every 4 days.

### Western blot analysis

Cell lysates were prepared by extracting proteins with lysis buffer [40 mM Tris-HCl (pH 8.0), 120 mM NaCl, 0.1% Nonidet-P40] supplemented with protease inhibitors. Proteins were separated by SDS-PAGE, and transferred to a nitrocellulose membrane (Amersham, Arlington Heights, IL). The membrane was blocked with 5% non-fat dry milk in Tris-buffered saline, and incubated with primary antibodies for overnight at 4 °C. Then the blots were developed with a peroxidase-conjugated secondary antibody, and proteins were visualized by enhanced chemiluminescence (ECL) procedures (Amersham, Arlington Heights, IL), using the manufacturer’s protocol.

### Immunocytochemistry

Briefly, cells were fixed with 4% paraformaldehyde and permeabilized with 0.1% Triton X-100 in PBS. Following cell fixation, cells were incubated with the appropriate primary antibodies in a solution of PBS with 1% bovine serum albumin and 0.1% Triton X-100 at 4 °C overnight. Antibodies used were as follows: human anti-E-cadherin (mouse polyclonal antibody, 1:200), N-cadherin (mouse polyclonal antibody, 1:200), and -Fibronectin (mouse polyclonal antibody, 1:200). Staining was visualized using anti-rabbit or anti-mouse Alexa Flour 488 (Molecular Probes) antibodies and nuclei were counterstained using 4, 6-diamidino-2-phenylindole (DAPI; Sigma). These cells were visualized with a fluorescence-microscope (Olympus IX71).

### Measurement of ROS Generation

To determine the amount of intracellular ROS in the mammary cells, cells were incubated in 10 μM 2, 7-dichlorodihydrofluoresceindiacetate (DCFH-DA; Invitrogen) at 37 °C for 15 min and washed with cold PBS three times and immediately retained DCF was analyzed by flow cytometer (BD FACS Calibur™) using Cell Quest software.

### Statistical analysis

All experimental data are reported as the mean ±standard deviation (S.D.) of at least three independent tests. Statistical analysis was performed by the parametric Student’s *t*- test to check the significance levels.

## Additional Information

**How to cite this article**: Kim, R.-K. *et al.* Beneficial effects of low dose radiation in response to the oncogenic KRAS induced cellular transformation. *Sci. Rep.* **5**, 15809; doi: 10.1038/srep15809 (2015).

## Supplementary Material

Supplementary Information
