# Supplementary material for: Beneficial effects of low dose radiation in response to the oncogenic KRAS induced cellular transformation
Source: Sci Rep. 2015 Oct 30;5:15809. doi: 10.1038/srep15809 (PMC4626770; doi:10.1038/srep15809)
Supplement: Supplementary Information [file srep15809-s1.pdf]

Supporting Information

Beneficial effects of low dose radiation in response to the oncogenic KRAS induced cellular transformation

Rae-Kwon Kim[1†], Min-Jung Kim[2†], Ki-Moon Seong[2†], Neha Kaushik[1], Yongjoon Suh[1], Ki-Chun Yoo[1], Yan-Hong Cui[1], Young-Woo Jin[2], Seon-Young Nam[3*], Su-Jae Lee[1*]

[1]*Department of Life Science, Research Institute for Natural Sciences, Hanyang University, Seoul, Korea*

[2]*Laboratory of Radiation Exposure and Therapeutics, National Radiation Emergency Medical Center, Korea Institute of Radiological and Medical Sciences, Seoul, Korea.*

[3]*Radiation Health Institute, Korea Hydro and Nuclear Power Co. Ltd, Seoul, Korea.*

Corresponding author email: sj0420@hanyang.ac.kr or synam6660@khnp.co.kr

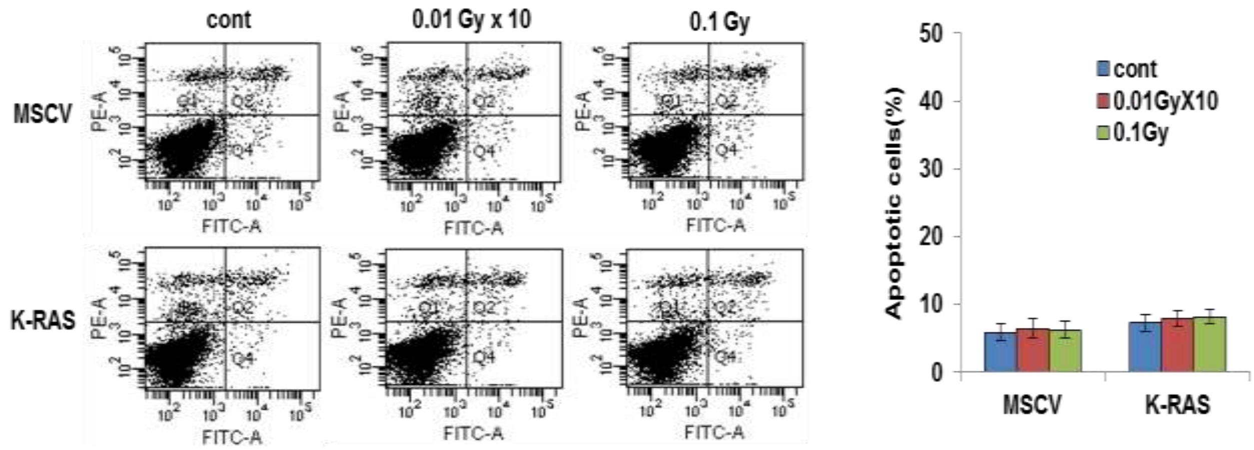

Figure S1. MCF10A Cells which were transfected with MSCV-KRAS or control empty MSCV vector, were either treated with 0.1 Gy at once or exposed with 0.01 Gy for 10 times (accumulative dose of 0.1 Gy) by ionizing radiation. Cell death was analyzed by Annexin V-FITC/PI staining using flow cytometry.

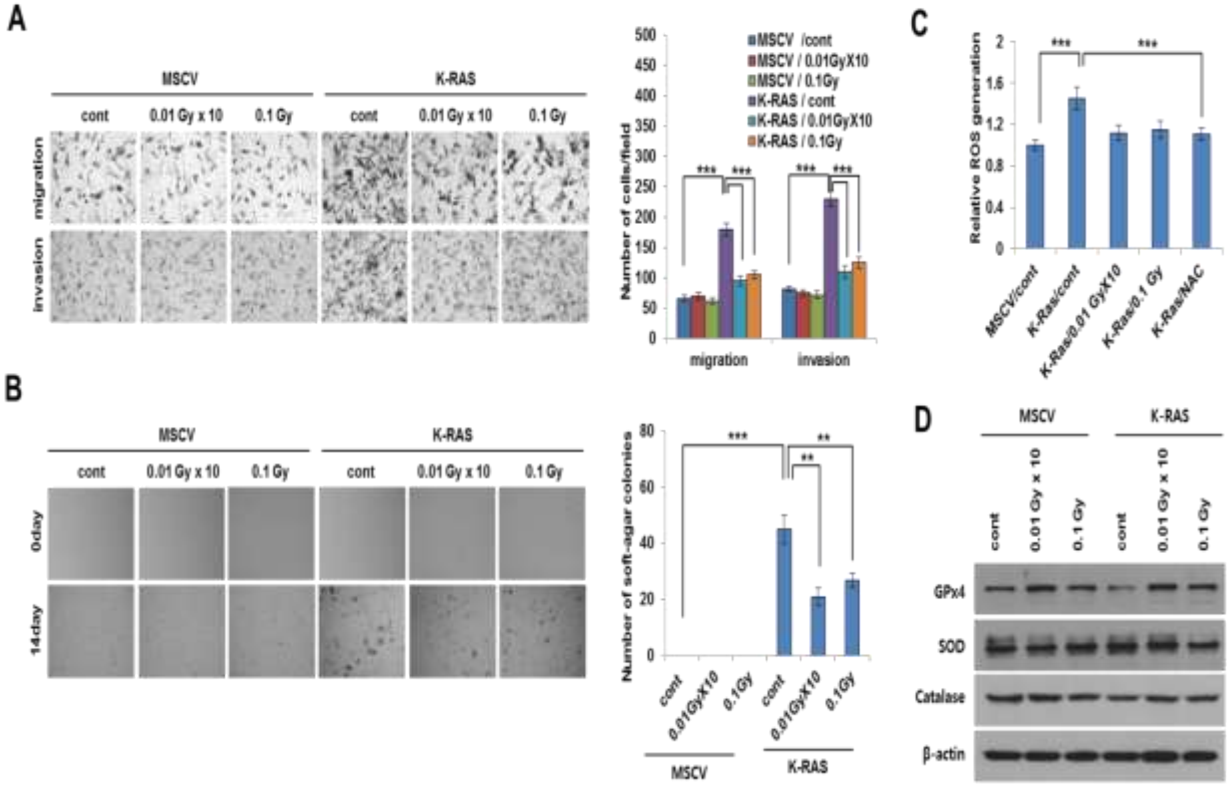

Figure S2. (A) Images and graphical representation of migration and invasion assay of MSCV-KRAS and control empty MSCV vector transfected low-dose irradiated-WI38 cells (Normal embryonic lung fibroblast cell line). (B) Soft agar assay of low-dose irradiated-WI-38 cells which were transfected with MSCV-KRAS and control empty MSCV vector. (C) Relative ROS generation of low-dose irradiated-WI38 cells which were transfected with MSCV-KRAS or control empty MSCV vector, as measured by flow cytometry. NAC was used as a ROS inhibitor to check the effect of low dose radiation as positive control. (D) Western blot analysis of antioxidants using cell lysate from low dose irradiated-WI-38 cells which were transfected with MSCV-KRAS and control empty MSCV vector.

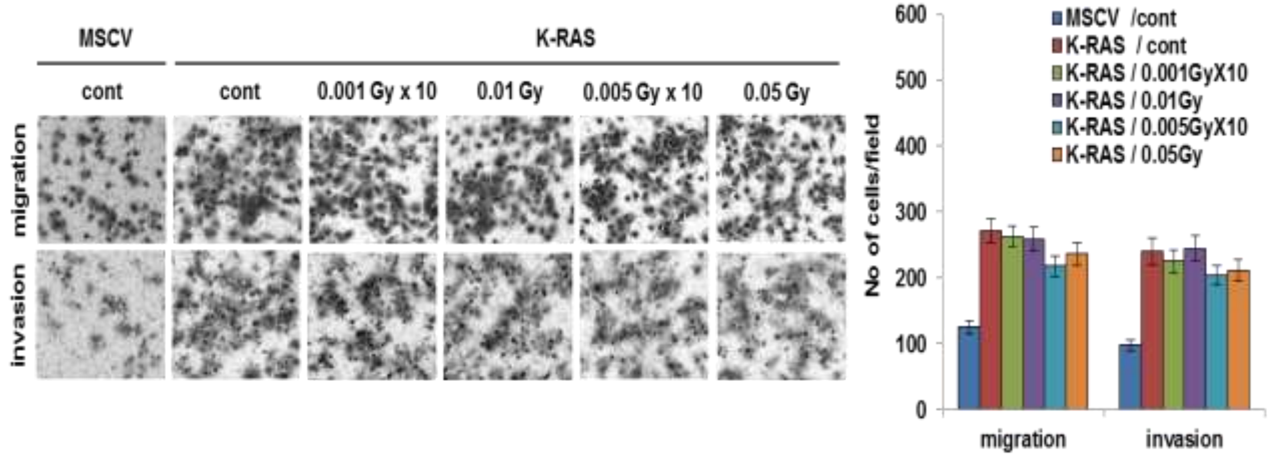

Figure S3. Images of migration and invasion assay of MSCV control or MSCV-KRAS transfected low dose radiation-treated MCF10A cells at a dose rate of 0.01 and 0.05 Gy (single and fractionated).

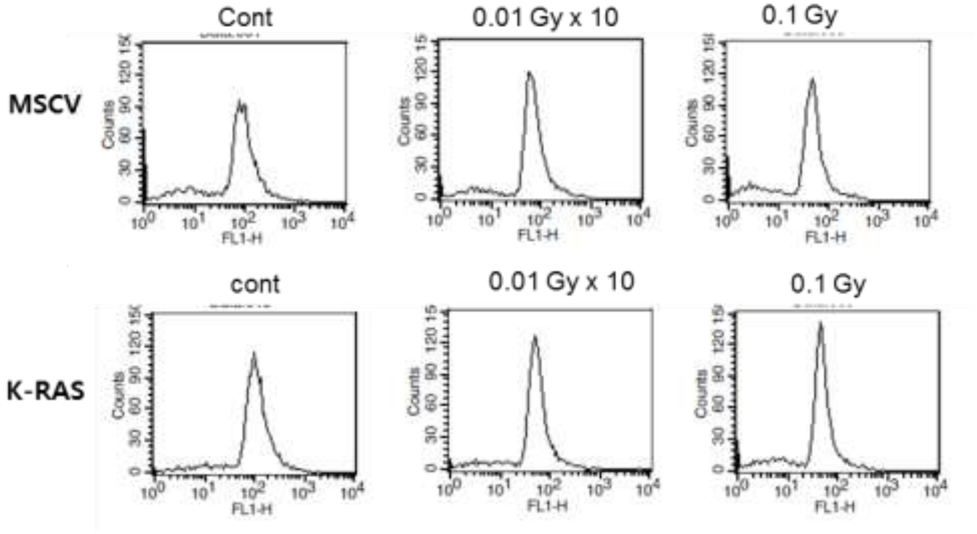

Figure S4. Cells were either treated with 0.1 Gy at once or exposed with 0.01 Gy for 10 times (accumulative dose of 0.1 Gy) by ionizing radiation. Relative ROS generation of low dose irradiated-MCF10A cells which were transfected with MSCV-KRAS or control empty MSCV vector, as measured by flow cytometry.

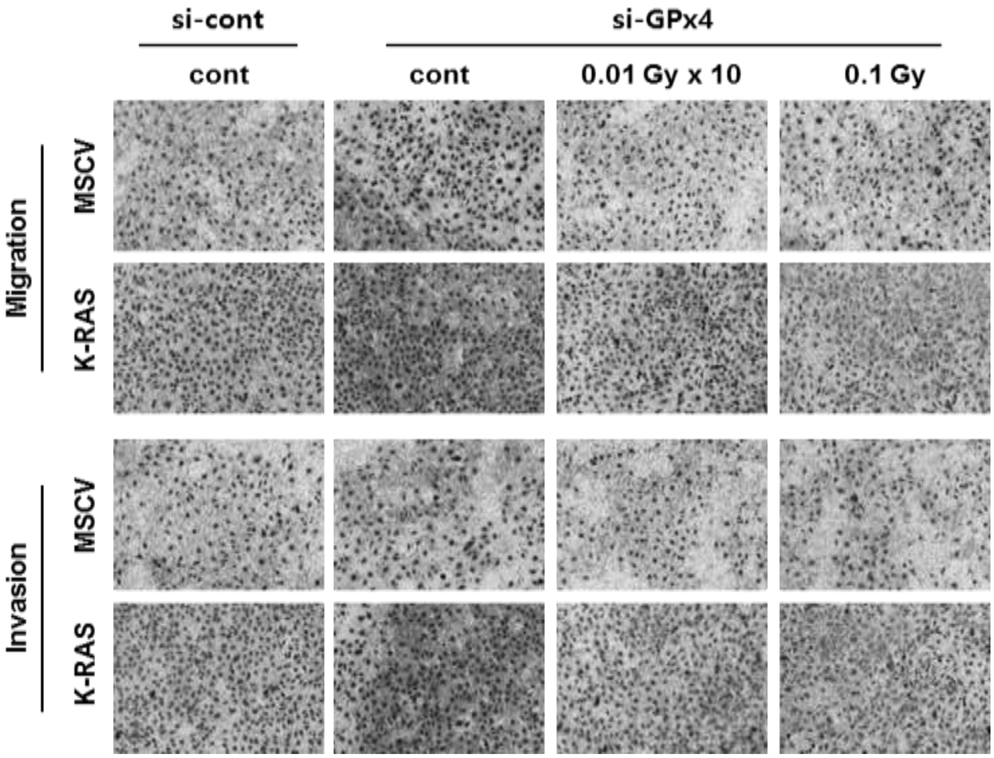

Figure S5. Images of migration and invasion assay of MSCV control or MSCV-KRAS transfected low dose radiation-treated MCF10A cells after blocking GPx4 expression using siRNA targeting.

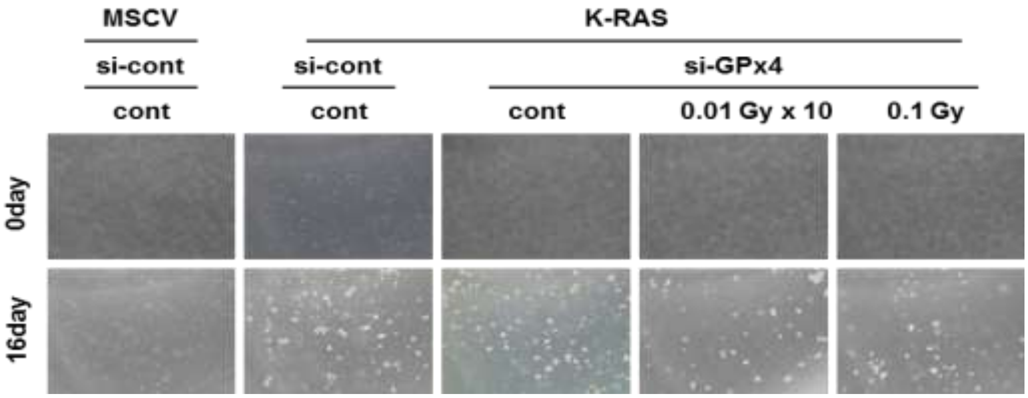

Figure S6. Photographic representation of number of soft agar colonies of MSCV control or MSCV-KRAS transfected low dose radiation-treated MCF10A cells after targeting of GPx4 expression using siRNA targeting after 16 day of treatment.
